# Multi-Sensors System and Deep Learning Models for Object Tracking

**DOI:** 10.3390/s23187804

**Published:** 2023-09-11

**Authors:** Ghina El Natour, Guillaume Bresson, Remi Trichet

**Affiliations:** 1 Continental, 1 Av. Paul Ourliac, 31100 Toulouse, France; remi.trichet@gmail.com; 2 Vedecom, 23 bis All. des Marronniers, 78000 Versailles, France; gubresson@gmail.com

**Keywords:** multi-sensors system, tracking, recurrent neural networks, sensor fusion, metric learning

## Abstract

Autonomous navigation relies on the crucial aspect of perceiving the environment to ensure the safe navigation of an autonomous platform, taking into consideration surrounding objects and their potential movements. Consequently, a fundamental requirement arises to accurately track and predict these objects’ trajectories. Three deep recurrent network architectures were defined to achieve this, fine-tuning their weights to optimize the tracking process. The effectiveness of this proposed pipeline has been assessed, with diverse tracking scenarios demonstrated in both sub-urban and highway environments. The evaluations have yielded promising results, affirming the potential of this approach in enhancing autonomous navigation capabilities.

## 1. Introduction

Nowadays, autonomous vehicles (AVs) are gradually becoming part of our lives. Autonomously navigating robots have been developed since 1961 when James Adams first developed an autonomous lunar rover during his Ph.D. Over the years, various autonomous robots and aircraft were developed, and Tesla launched Autopilot in 2015. Nowadays, Waymo driverless taxi cars are employed in Arizona, USA. 

However, the path to developing autonomous vehicles has not been without challenges. Back in 2010, manufacturers such as Honda, Tesla, and Toyota claimed that, by 2020, fully autonomous vehicles would be operational, and users would be sitting in the back seat. By 2023, it became evident that this ambitious objective faced multiple challenges, particularly concerning safety. Although recent studies [1] claim that cars equipped with ADAS (Advanced Driver Assistance Systems) technology experience a 23% reduction in crash frequency, fully automated vehicle safety remains a significant concern. On March 18, 2018, the first driverless deadly accident occurred when an Uber autonomous vehicle hit and killed a pedestrian walking by her bicycle in Arizona, USA. According to the National Transportation Safety Board accident report, the near-range sensor was not correctly operational and failed to predict the object’s movement because it was not at a crosswalk. Following this incident, multiple AV accidents have been reported, serving as a reminder that more work should be brought to sensor technology and object tracking for autonomous vehicles.

Autonomous vehicles rely on an array of sensors, including cameras, radars, lidars, …, to perceive their surrounding environment. At each time step, noisy sensor detections are received and treated to identify the surrounding objects and their potential trajectories. In this paper, we are interested in the fusion of two hybrid sensors at the core of the most prevalent association: vision and radar. The complementarity of these sensors makes their fusion a natural choice. 

**Figure 1:**
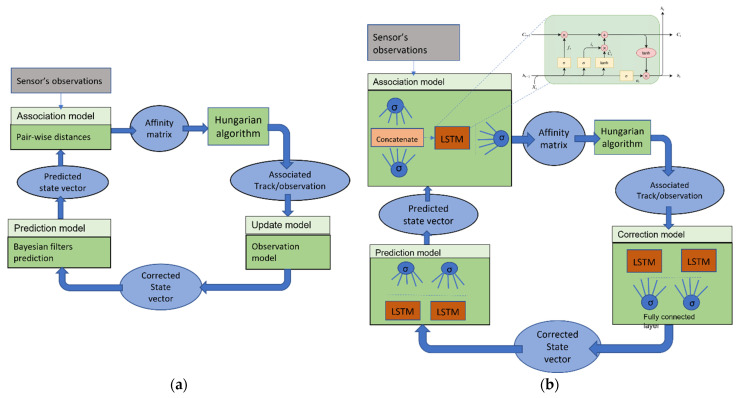
An illustration of the object-tracking process. (**a**) Bayesian-based approach. (**b**) Our approach consists of replacing classical algorithms to solve the association, prediction, and update problems by LSTM-based algorithms.

The goal is to track objects in front of an AV. Figure 1 illustrates the sequential steps involved in the object-tracking process. Our approach consists of replacing classical algorithms that tackle association, prediction, and update problems (Figure 1a) with recurrent neural networks (RNN)-based algorithms. RNNs are chosen for their ability to learn the dynamic properties of moving objects, but the scarcity of annotated data poses a challenge for LSTM-based (Long-Short Term Memory) [2] tracking techniques. The problem is divided into multiple tasks to overcome this, and simple models are trained to solve those tasks, as illustrated in Figure 1b. 

In the Bayesian filter methodology, the object state vector is updated based on previous estimations and the received observations. Authors in [3] and [4] showed that the Kalman filter works better on the sensor error than on the sensor output. Building on this idea, we incorporate a single layer of an LSTM-based model that learns the observation error model for each sensor. At each time stamp, we predict the observation error using this model to refine and correct the observations accordingly.

Furthermore, in the proposed association method, we want to learn an association metric that is more representative than Euclidian distance. Indeed, the Euclidian distance is generally applied to compute the association metric, but it is not fully representative of the object state because it only considers the object’s position (2D coordinates). The learned association metric considers the object state vector, including (x,y) position, class type, and orientation. To address the problem of the time-varying number of objects, especially using neural networks involving a fixed input size, the association model evaluates one combination at a time, and then a classic Hungarian algorithm loops over all combinations to find the best correspondences.

Finally, we propose a new existence score computation aimed at managing the track’s lifecycle. The existence score of a track is crafted to encompass its association scores with both sensor observations as well as the count of skipped frames. We want to take advantage of the association probability estimated using the association model because this information allows us to automatically account for whether a track is associated with one or two sensor observations. This approach serves the dual purpose of managing the initiation and termination of a track when it enters or exits the sensor’s field of view. Secondly, to eliminate false positive tracks originating solely from sensor-only observations.

In this paper, we propose a novel LSTM-based tracking algorithm that addresses several challenges in object tracking for autonomous systems and that performs the following:

- Leverages the need for a large amount of annotated data by dividing the tracking problem into multiple tasks and utilizing single-layer models trained for the association, the prediction, and the update steps.
- Improve the accuracy of objects’ positions by estimating the observation errors rather than direct position estimation, utilizing the updated model.
- Learning an optimized association metric by combining diverse cues such as object position, type, orientation, and trajectory into a unified model that estimates the association score for an observation/track pair.
- Manage tracks by computing a variable existence score that incorporates association scores, participating sensors, lifetime, and track disappearance.

### State of the Art

In this paper, we are interested in the fusion of two hybrid sensors for object tracking that nest within the most common sensor association: vision and radar. The complementary nature of these sensors has inspired previous approaches [5]. Wang et al. [6] utilized radar detections to guide object search in images, while Yu et al. [7] presented a high-level fusion of camera and radar sensors, leveraging deep learning for object detection and Kalman filtering for tracking. Although recent research tends to favor the elimination of radar sensors in favor of relying solely on camera sensors for advanced and autonomous driving functions, as mentioned by the authors in their review [8], it is important to note that camera sensors still have significant limitations, particularly in the distance and velocity estimation. To address these limitations and ensure better precision and completeness of detections, the integration of range sensors remains necessary.

Joly et al. [9] conducted a comparative study of several nonlinear filters and found that the particle filter offers enhanced robustness compared with the Kalman filter, though the number of particles affects both performance and computational costs. One limitation of Bayesian filtering is the necessity to model the behavior of the objects being tracked, which may not accurately reflect the real movements of different object types, such as pedestrians, vehicles, or cyclists. 

Since 2010, the rise of deep learning (DL) has led to groundbreaking advancements in autonomous vehicles and object tracking. RNNs successfully applied to language modeling by Wang [10] set the foundation for progress in this domain. In particular, LSTM networks have become the most studied type of RNNs due to their ability to overcome the vanishing gradient problem through multiple gates controlling the network’s input/output status and memory. LSTMs’ model-free nature allows them to model both linear and nonlinear dynamics, effectively capturing object motion patterns through trajectory analysis. Comparatively, Bayesian filters require substantial efforts in designing state transition and measurement models, making LSTMs a favorable alternative, as noted by Iter [11], Gu [12], and Altché [13]. Ondruska [14] demonstrated the versatility of RNNs in multi-object tracking by directly mapping raw radar sensor inputs to object tracks in the sensor space, eliminating the need for formal data association. Further research has explored LSTM’s predictive capabilities for trajectory estimation of pedestrians, Alahi [15], and vehicles, Ma [16]. Park [17] introduced an LSTM encoder-decoder architecture designed for vehicle trajectory prediction. Their model takes as input the positional and velocity data of the ego vehicle as well as that of surrounding vehicles. The model’s output consists of an occupancy grid map containing the k most probable trajectories for each vehicle. However, the beam width K is manually set to a value of 10, thereby introducing heightened complexity into their algorithm. In their work, Sengupta [18] introduced a sensor fusion approach that leveraged deep neural networks and LSTMs to integrate data from a monocular camera and a mmWave radar. Both sensors’ object positions are extracted in 2D coordinates, with subsequent utilization of the LSTM-based model for predicting object trajectories. However, in their study, the sensor-only tracks (tracks detected by only one sensor) were not effectively managed, which led to an increased risk of false positives over time. Moreover, their fusion methodology primarily yielded a fused position comprising the lateral coordinates from the camera and the longitudinal coordinates from the radar rather than providing a refined representation of the sensor’s observations. This discrepancy is addressed and rectified in our approach through the application of the LSTM correction model. Da Zhang [19] presented a significant breakthrough with the Deep RL Tracker (DRLT) algorithm, which combines Convolutional Neural Networks (CNNs), RNNs, and RL to achieve precise object tracking in videos. While showing good performance compared with state-of-the-art algorithms, DRLT requires the initial ground truth location for each object and leaves room for addressing the association problem.

Data association in multi-sensor-based tracking is a significant challenge, involving multiple objects and detections received at each time step, with varying numbers over time and potential false positives. Addressing this problem entails two key steps: metric learning and affinity matrix optimization. The affinity matrix contains similarity scores obtained by comparing cues, such as motion and appearance, between track/observation pairs. The goal is to achieve an optimal assignment of tracks to observations. The Hungarian algorithm, also called Global Nearest Neighbor (GNN) [20,21], is a well-known and widely used approach to solve the bipartite graph problem of affinity matrix optimization. Additionally, Multiple-Hypothesis Tracking (MHT) [22] and Joint Probability Data Association (JPDA) [23] are considered to outperform the GNN in clusters but become impractical for real-time settings as the number of objects increases. 

Methods addressing the association metric can be categorized into two approaches. The first approach involves computing pair-wise distances between sensors’ measurements and state predictions. The second approach utilizes deep learning algorithms, combining multiple cues such as appearance and dynamic cues. Milan [24] presented an online pedestrian tracking approach using RNN. They performed prediction, data association, and state updates within a unified network. The association vector was computed using an LSTM-based network, utilizing Euclidean distance between the predicted state and the observations as input. Similarly, Liu [25] designed an LSTM network to learn the measurement-to-track association probability from radar noisy measurements and existing tracks. The input to the LSTM network is also a pair-wise distance matrix generated from all measurements and state prediction of each target without explicit metric learning. Using the Euclidean distance as an association metric might not accurately reflect the track-to-object correspondence as it only considers coordinates parameters. Building on this, Sadeghian [26] utilized hierarchical RNNs to encode multiple cues, including appearance, motion, and interaction, for offline metric learning. Following a similar line, Wan [27] fused three features (appearance, motion, and velocity) into one LSTM network for metric learning. The Hungarian algorithm is employed to solve the global optimization problem. Luo [28] concatenated motion features to appearance features extracted by a CNN. However, in certain scenarios, the appearance of objects is not available; sensors such as radar or lidar provide very limited appearance information. Huang [29] introduced a straightforward tracking-by-detection framework, achieving good performance on the BDD100K MOT and MOTS datasets. Their main contribution is in the association step of objects bounding boxes across frames. However, they discard motion information, relying solely on appearance embeddings for object association. Kieritz in [30] proposed to perform joint detection and association in a unified model. The input cues for the association layer are spatial distance, object appearance, and detection and tracking score. The association score matrix is then optimized using the Hungarian algorithm. In their work [31], Yoon introduced an encoder-decoder model with bidirectional LSTM to calculate an association affinity matrix. This model takes non-appearance features as input, including bounding box coordinates and detection confidences. The optimization of the association matrix was achieved using the Hungarian algorithm. Similarly, we do not use appearance cues as input to our association model. Nevertheless, our model is considerably simpler and requires less data for training.

In this paper, we aim to provide new insights into sensor fusion for object tracking, seeking to overcome traditional Bayesian filter limitations and achieve improved performance and accuracy in various tracking scenarios. The remainder of this paper is structured as follows. Section 2 details the proposed multi-sensor system for object tracking. Section 3 evaluates the proposed system experimentally. Finally, Section 4 summarizes and concludes the paper.

## 2. Materials and Methods

### 2.1. The Sensors 

Our study utilizes a multi-sensor system, including radar and a camera with overlapping fields of view, to track objects in front of an autonomous truck. Additionally, an RT range sensor serves as a ground truth reference. We collected a database in highway and peri-urban environments, with sensors triggered at 10 frames per second. The raw data are prepossessed, and objects are detected. The state vector of each sensor observation is provided, including its position, type, and orientation angle.

### 2.2. Problem Formalization

The sensor’s observation set at time *t* is defined as follows: $O_{t}^{k}={\left\{o_{1}\ldots o_{N}\right\}}_{t}^{k}\;$ where *N* is the number of observations of sensor *k.* Each observation is represented by a state vector containing the position, the velocity, the orientation, etc. 

A tracked object is identified by a unique identifier, a state vector, and a trajectory which is composed of the state vectors from *t0* to *t1.* The track set is defined as follows: $X_{t}={\left\{x_{1},\ldots ,x_{M}\right\}}_{t}\;$ where *M* is the number of simultaneous tracked objects.

**Figure 2:**
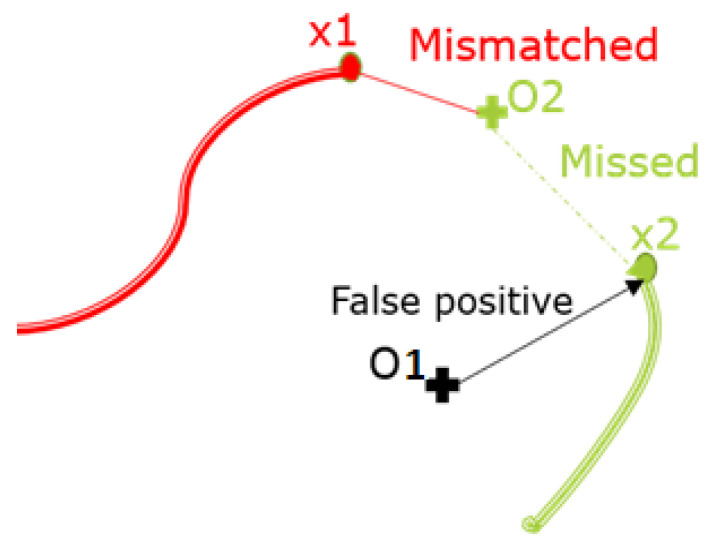
Illustration of association ambiguities. O stands for observations and x stands for the objects.

The goal is to continuously track objects and accurately estimate their positions at each timestamp. For this, the future position of a track at the next timestamp needs to be predicted to associate it with the received observation. Therefore, each track is associated with at least one observation if it is still visible by the sensors. However, data association poses significant challenges due to the variable cardinality of objects and observations at each timestamp. Ambiguities in association can arise from occlusion or association problems (misses, mismatches, and false negatives), as illustrated in Figure 2.

Once associated, the predictions are then corrected with the observations. We want to avoid the modeling step because the models may not reflect the correct dynamic of the objects or the correct sensor observations. Thus, we proposed three deep learning models to handle the prediction, association, and update steps for object tracking.

### 2.3. The Models

The proposed models are built upon LSTM recurrent neural networks. The inclusion of recurrent neural networks allows us to capture the intricate interdependencies present in sequences of temporal data, such as object trajectory sequences in our context. The term “recurrent” stems from the feedback loop that is passed through the iterations, enabling the network to retain and process information over time. To optimize the network’s performance, we empirically set the hidden layer size to 200, ensuring a balance between complexity and efficiency.

#### 2.3.1. Association Model

**Figure 3:**
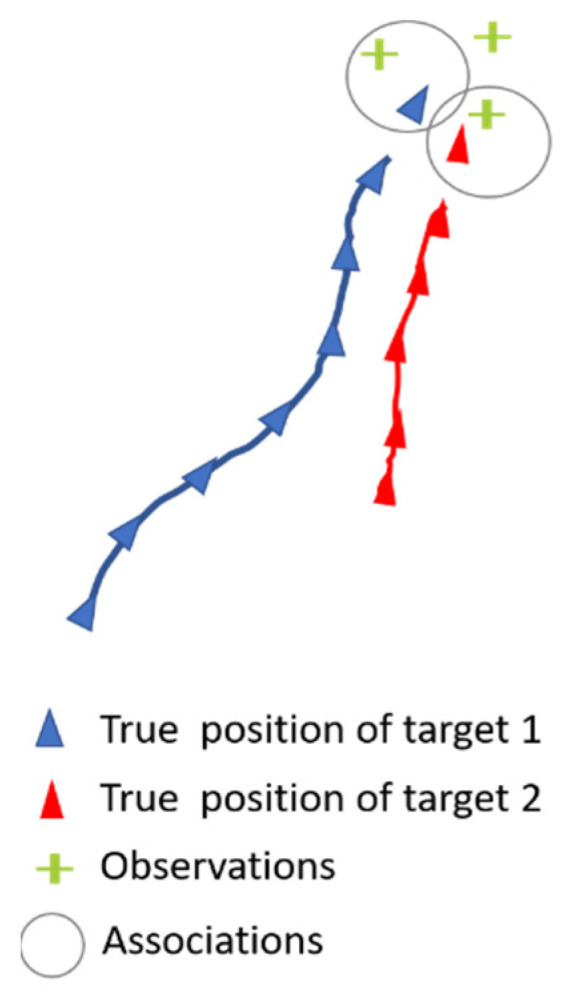
Scene illustration with 2 objects and 3 observations at time *t.* The circles depict the combinations to be evaluated.

Our primary focus is on solving the data association between tracks and observations received at time *t*. Figure 3 illustrates an example of a scene at time *t* with two objects and three sensors’ observations. To achieve this, we employ an association model that evaluates all possible combinations of object/observation associations. The model takes an object’s state vector sequence from time $t_{0}$ to time t−1 and concatenates it with an observation obtained from one of the sensors at time t.

**Figure 4:**
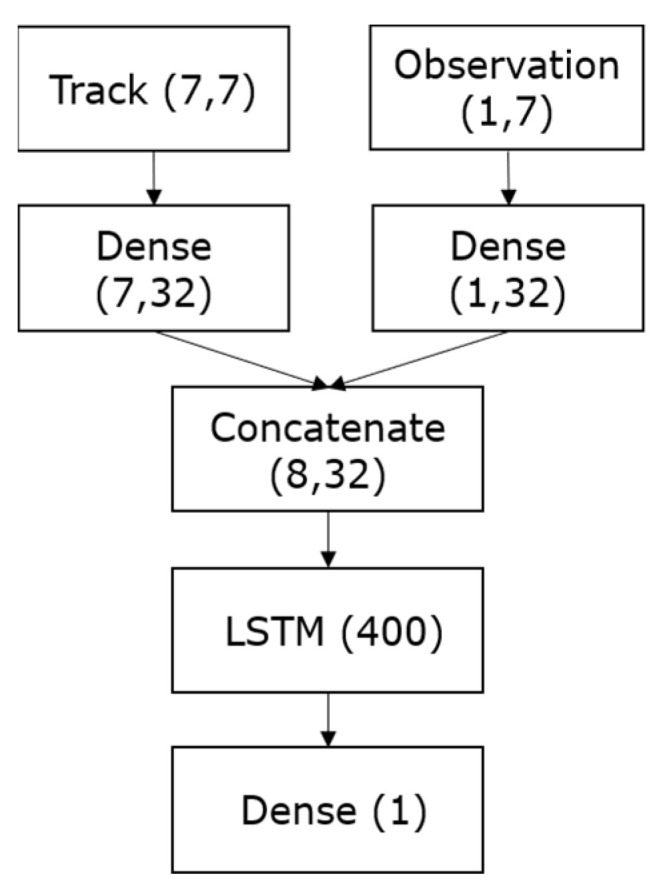
The association model architecture.

An LSTM layer of hidden size set to 400 is followed by a fully connected dense layer with only one neuron and a sigmoid activation function, which makes it possible to output a probability of association in the range [0, 1], indicating the likelihood of an association. During training, we employ the logarithmic loss function (binary cross entropy) commonly used for binary classification problems. Hence, the LSTM-based feature extractor is learned by minimizing the binary cross entropy (BCE) loss, formulated as follows: $L_{BCE}=-$($\underset{n=i}{\sum }y_{i}\;\log\left(f\left(s_{i}\right)\right)+\left(1-y_{i}\right)\log\left(1-f\left(s_{i}\right)\right)$), where *s* is the input, f is the activation sigmoid function, y is the correct classification, and i is the class to predict. The architecture of the association model is depicted in Figure 4 below.

#### 2.3.2. Correction Model

**Figure 5:**
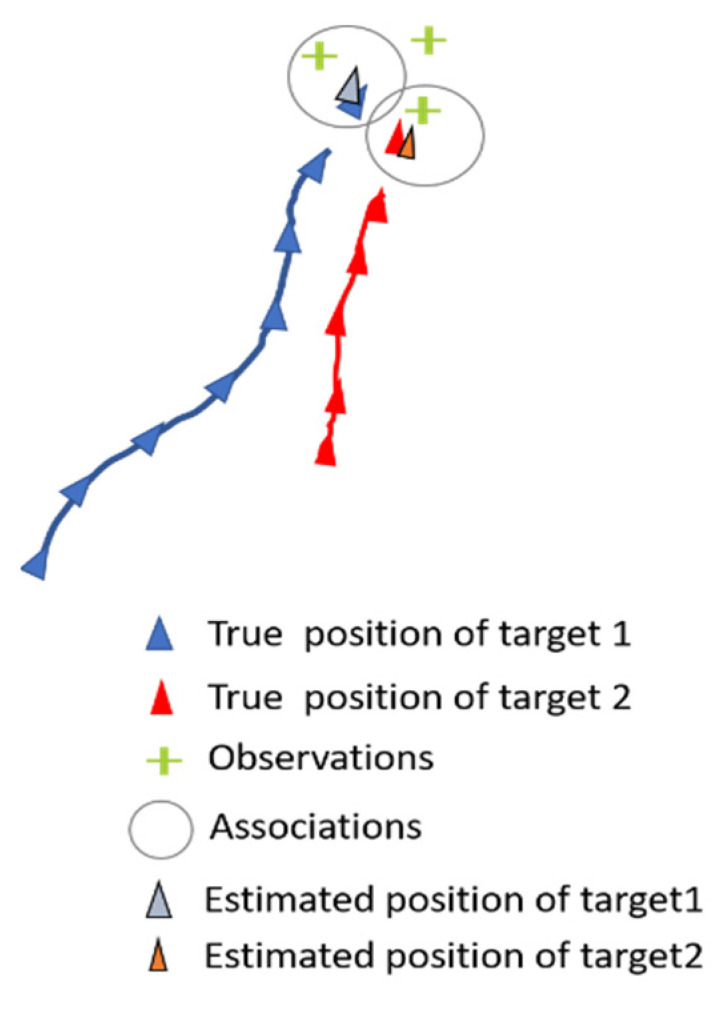
Scene illustration with two tracks and three observations already associated and the observations corrections at instant *t*.

The goal of the correction model is to replace the update step of a Kalman filter, brought in to correct the Kalman predictions using the received observations and the sensor’s observation models. Figure 5 illustrates the scene with two objects already associated with two observations out of three, and the observation positions are corrected (estimated positions using the correction model). 

**Figure 6:**
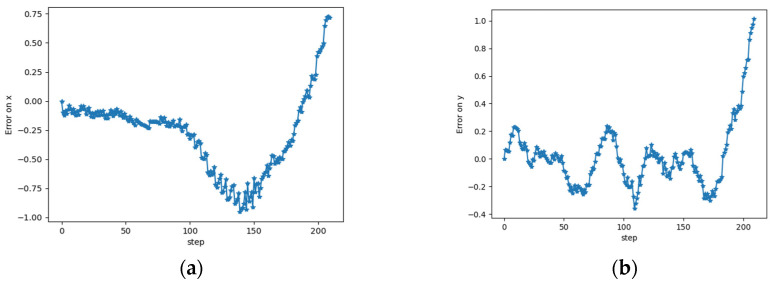
Observation error plots on x(m) (**a**) and y(m) (**b**) coordinates.

The state vector of a moving object at a given time *t* depends on the previous state vector at *t−1*. The correction model is designed to predict the observation’s errors in an instant, *t* having as input an observation error sequence shown in Figure 6. For the training, the observation error at each time instant is computed by comparing the sensors’ observation to the ground truth positions. 

**Figure 7:**
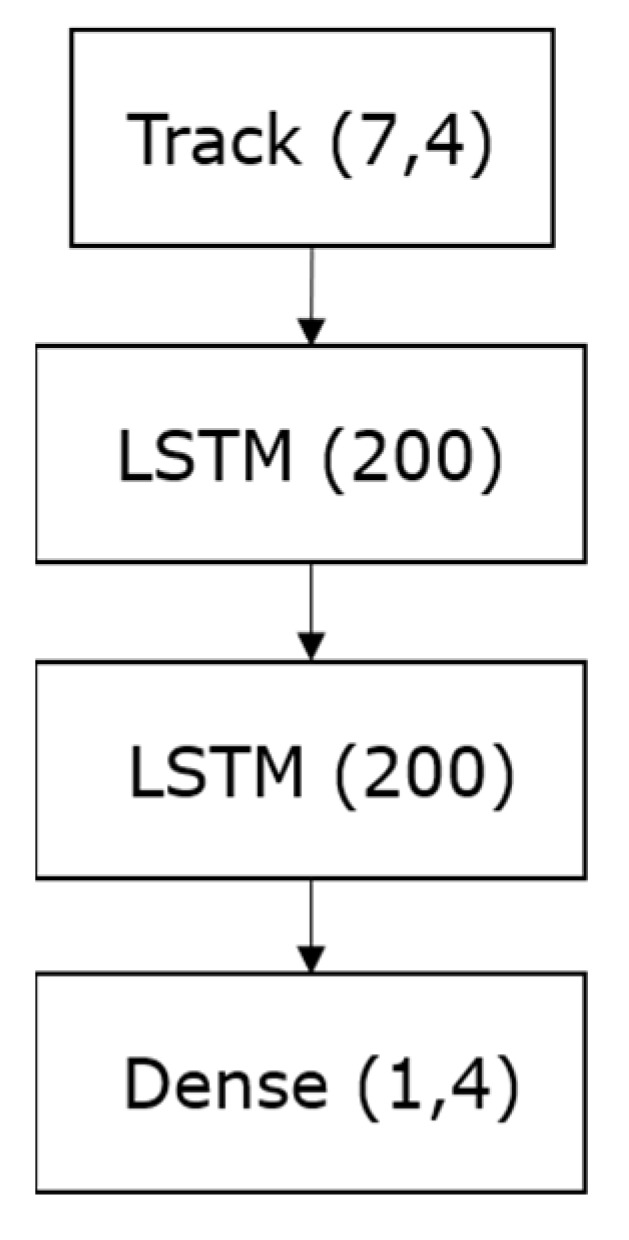
The update model architecture.

The correction model utilizes the observation’s error sequence as input, which is then passed through two LSTM layers, each with a hidden layer size of 200. Subsequently, a fully connected dense layer is applied with a sigmoid activation function to predict the next observation’s errors. The model architecture is depicted in Figure 7.

The learning is done by optimizing the MSE (Mean Squared Error) loss function for prediction:

$L_{MSE}=\frac{1}{N}\;\overset{N}{\underset{i=1}{\sum }}\;{\left|\left(Y_{t}^{i}-{\hat{Y}}_{t}^{i}\right)\right|}^{2}$, where $Y_{t}^{i}$ is the correct observation error at *t* and ${\hat{Y}}_{t}^{i}$ is the estimated observation error at *t*. 

#### 2.3.3. Prediction Model

To ensure accurate track-to-observation association, we want to predict the object’s position at the same time instant as the observation. For this purpose, the same correction model architecture is employed to predict the next position from a sequence of object trajectories. The input to the model is the track trajectory sequence starting from time *t*_0_ up to the current instant *t*. The model then processes this trajectory sequence and produces the prediction of the object’s state at time *t* + 1. 

Similarly to the correction model, the MSE loss function is optimized for prediction: $L_{MSE}=\frac{1}{N}\;\overset{N}{\underset{i=1}{\sum }}\;{\left|\left(Y_{t}^{i}-{\hat{Y}}_{t}^{i}\right)\right|}^{2}$, where $Y_{t}^{i}$ is the correct position at time t and ${\hat{Y}}_{t}^{i}$ is the estimated position at time t. 

#### 2.3.4. Models Training

We trained the three models using a database of 10 diverse sequences with a total running time of approximately 12 min. Trajectories were divided into training and testing sets, including cars, trucks, motorcycles, and pedestrians, with varying speeds between 0 and 80 kph. The ego vehicle speed varies from 20 to 80 kph.

We extracted 5000 trajectory sequences of length = 7 time steps from the ground truth objects trajectories. Additionally, the 8th state vector from the ground truth sequence is used as the output for the prediction model training. The computational complexity of the LSTM depends on the depth of the model and the sequence length, and because we chose simple models’ architectures, the complexity of the network is roughly dependent on the sequence length. Thus, the sequence’s length was chosen empirically in a way that it is not too short to have significant information for the model to learn and not too long, so it will not make the training too hard. The radar and camera observations errors corresponding to the trajectories were computed and used to train the update model. Finally, the correct association probabilities (0 or 1) are generated to train the association model. 

The extracted data was scaled to a range of [0, 1] to ensure uniformity in the data with different scales, aligning with the sigmoid activation function of the units in the final layer responsible for the predictions. For optimization during training, we opted for the ADAM optimizer, known for its faster convergence and stability compared to other optimizers, according to this study in [32]. We chose a learning rate of 0.001 based on our experiments, which falls within the optimal range of 0.001 to 0.01 recommended in the previous study. The models were trained for 300 epochs, iteratively refining their performance and enabling them to make more accurate predictions. 

### 2.4. Track Management

Track management plays a vital role in the tracking process as it handles object birth and death while dealing with association ambiguities. An existence score is computed for each tracked object based on its association scores with radar and camera observations to achieve this, along with the number of skipped detections.

The existence score (*ES*) is calculated as follows:

(1) $$ES=RAC_{i}+CAC_{i}-SF$$

Here, $RAC_{i}$ and $CAC_{i}$ are the radar association cost and camera association cost for object *i*. The existence score increases when an object has a high association probability with an observation.

To mitigate false births caused by noisy detections, a validity threshold is set. When a new object is detected, its state is initially set to ‘hidden’ until it consecutively appears in the next iterations, at which point its state is changed to ‘visible.’ The number of skipped frames (*SF*) for an object is incremented by 1 if the track is not observed by any sensor (not associated with any observation) and by 0.5 if it is observed by only one of the sensors. Consequently, the existence score decreases when the object is not observed by one or both sensors.

**Figure 8:**
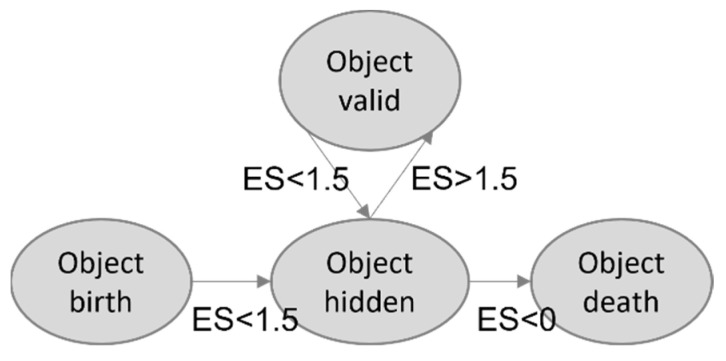
Illustration of an object’s life state.

If a track is no longer detected, its state is set to ‘hidden,’ and it is propagated into the future by predicting its next position to handle object occlusion. Finally, when the existence score falls below the death threshold, set to 0, the track is deleted. Figure 8 illustrates the various states a track can pass through during its lifetime, demonstrating the dynamic nature of the track management process.

## 3. Evaluation and Results

To gain deeper insights into the performance of the proposed algorithms, we conducted evaluations of a multi-object tracker using various types of sequences available in the dataset. Specifically, we considered scenarios involving heading targets and lane changing in highway environments, as well as oncoming vehicles in peri-urban environments.

The truck utilized in the evaluation is equipped with an ARS430 radar sensor with a lateral field of view of ±60°. The range resolution is 2.23 m, while the azimuth resolution is 3.2° at 0° and increases for angles below 0°. The radar provides a range accuracy of approximately ±0.55 m and ±0.1° on azimuth at 0° for the far scan. In addition, an MFC520 camera is used. Objects are detected within the camera’s field of view and classified within a detection range of up to 150 m. The lateral accuracy of the MFC is approximately ±0.5 m, while the longitudinal accuracy varies, being ±0.5 m for near objects and higher for farther objects.

**Figure 9:**
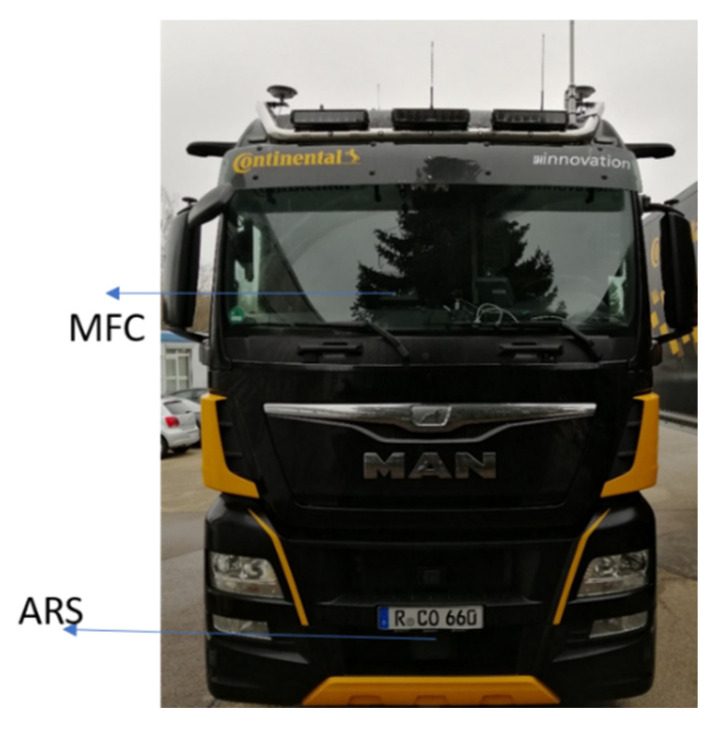
The setup of the sensors (MFC520 and ARS430) mounted on a truck for training and test collection data.

Figure 9 illustrates the mounting setup of the sensors (MFC520 and ARS430) on the truck, which is also equipped with an RT-range (real-time) system from OXTS used as a ground truth for object positions. The RT system measures the relative positions between an RT inertial system and other mobile targets equipped with RT-range systems. The accuracy for object range measurements is 0.03 m. The sensor observations are transformed into a common frame relative to the ego vehicle.

The evaluation database consists of 10 trajectories, corresponding to a running time of approximately 16 min. Both peri-urban and highway scenarios are included in the evaluation setup.

Unlike appearance-based methods, our proposed method does not rely on appearance cues; instead, we receive camera observations at the object level. As a result, comparing our method with appearance-based algorithms is challenging due to the black-box nature of the object detection algorithm employed for the MFC camera. As stated by Mouzaffari in [33], unlike object detection, which employs a standardized evaluation framework, the field of vehicle behavior prediction lacks a consistent benchmark. This absence of a benchmark hinders fair comparisons among various deep learning-based approaches and between deep learning-based methods and other techniques. To evaluate our approach, we compare the obtained results with those of an Extended Kalman-based tracking algorithm using the same database acquired by the same sensors. For the evaluation of the multi-object tracking algorithm, we use the following metrics:

- The RMSE (Root-Mean-Square Error) to evaluate object position estimation, calculated as the mean Euclidean distance over all predicted positions and real positions: RMSE= $\sqrt{\frac{1}{N}\;\overset{N}{\underset{i=1}{\sum }}\;{\left(Y_{t}^{i}-{\hat{Y}}_{t}^{i}\right)}^{2}}$, where $Y\;and\;\hat{Y}$ are the respective correct (RT ground truth) and predicted outputs.
- The MOTA (multi-object tracking accuracy) [34] to assess the association model and track management process: MOTA= $1-\frac{\sum_{b}\left(m_{t}+fp_{t}+mme_{t}\right)}{\sum_{t}g_{t}}$, where *t* is the frame index, and *m, fp*, and *mme* denote the number of misses, false positives, and mismatches, respectively, over *g*, the total number of objects in the scene across all iterations *b*. MOTA is a widely used metric as it effectively combines the three sources of errors mentioned above.

### 3.1. Results

**Figure 10:**
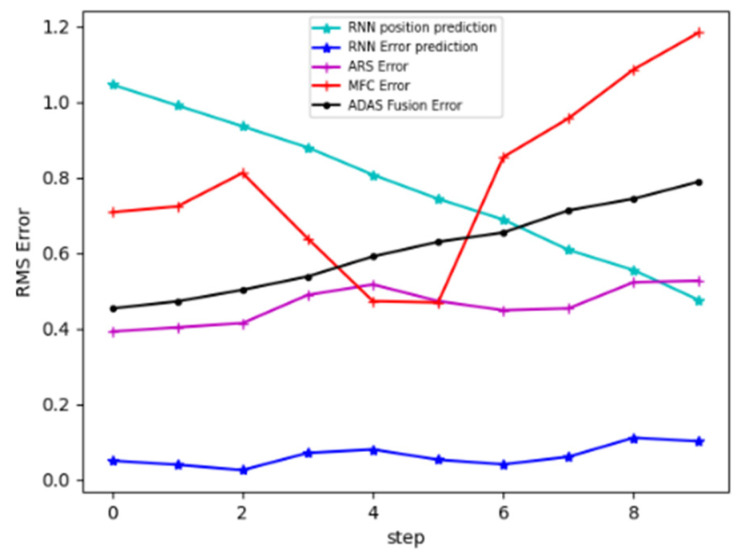
RMS errors (in m) graphs comparison on 10 timestamps of the sensors observations errors with the ADAS fusion error using Kalman filtering and the LSTM correction and prediction errors. The LSTM-corrected position error (in blue) is the lowest, with an average of 0.09 m.

To assess the accuracy of the tracking system, we compared the RMS position errors obtained from different sources with the ground truth provided by the RT-range system. For this evaluation, we extracted 1000 subsequences of length 10 from the test sequences. RMS errors were computed at each time step across the 1000 subsequences, considering various test scenarios such as cars on the ego lane, cars changing lanes, and two cars on adjacent lanes. The resulting RMS errors are plotted in Figure 10.

The MFC camera exhibited an error of approximately 0.91 m for objects at distances between 20 m and 60 m from the ego vehicle. In contrast, the radar observations had an average error of 0.5 m, while the LSTM correction achieved an average error of 0.09 m. Comparing this with an Extended Kalman filter-based algorithm, which had an average error of 0.7 m, the LSTM correction outperformed it by a factor of approximately 7.

Regarding the LSTM prediction error, it was approximately 1 m at the beginning of the sequence and decreased to around 0.47 m by the end of the sequence. This behavior indicates that the LSTM improved its prediction accuracy with longer sequences, capturing richer dynamic properties of moving targets. It is worth noting that the LSTM update error estimation was lower than the state prediction error, indicating that the LSTM-based method fits the sensor error model better than the object’s trajectory. To facilitate the training step, we divided the tracking problem into multiple stages and used simple model architectures for each step. Consequently, we did not require an extensive training dataset. Our proposed LSTM-based method is model-free, making it adaptable to various dynamic scenarios and sensor observations. However, it does require a training dataset with reliable ground truth for learning purposes. A comprehensive comparison between our method and the Kalman filter-based method is presented in Table 1, highlighting the advantages and efficacy of our LSTM-based approach.

Table 2 shows MOTA score equal to 95%. This score is derived from the combination of three types of errors: false positives (2.1%), misses (0.17%), and mismatches (2.6%). An important observation from the MOTA results is that the association algorithm tends to make more false associations than not associate observations at all. This behavior can be attributed to the limited occurrence of false positive observations compared with real positives in real-world scenarios, and this characteristic is reflected in the training data. Furthermore, as highlighted by Gioele [35], the MOTA score exhibits a significant correlation with the count of missed detections (false negatives). Notably, our algorithm has successfully minimized the number of false negatives, a result attributed to the inclusion of occluded tracks and sensor-only tracks within the hidden state.

One notable advantage of our trained model is that it exhibits a decrease of approximately 1.5% in mismatches error when compared with the Euclidean distance algorithm. This improvement is attributed to the fact that the Euclidean distance algorithm relies solely on the track’s coordinates, whereas our association model takes into account additional cues such as trajectories and object types, resulting in a more accurate association of observations. Additionally, contrarily to the association algorithm, the misses error for the Euclidean distance algorithm is affected by an empirically chosen distance threshold. This can impact the accuracy of the misses error, and the threshold may not adapt optimally to different scenarios.

**Figure 11:**
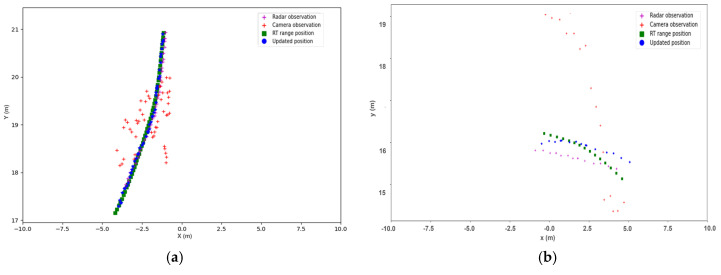
LSTM corrections (blue dots) for object trajectories with sensor observations (red and magenta crosses) and RT range (green squares). In (**a**), radar has a better estimation of object position than camera observation, and LSTM-corrected position best aligns with the RT ground truth. In (**b**), both sensors have notable observation errors, but LSTM-corrected positions are much closer to the ground truth compared with relying solely on the raw observations.

Figure 11 illustrates the LSTM correction results of object trajectories. The figure includes the ground truth positions and sensor observations. In Figure 11a, the radar observations closely align with the RT range ground truth (depicted by green squares) trajectories. However, it is evident that the camera observations are less precise along the longitudinal axis. The LSTM-corrected positions (indicated by blue dots) demonstrate the closest match to the RT ground truth. Figure 11b presents a scenario where both radar and camera observations exhibit high observation errors. Despite this, the LSTM correction manages to provide positions that are significantly closer to the ground truth than the raw observations alone. This demonstrates the LSTM’s capability to effectively learn and correct dynamic object behavior, specifically for cars in this scenario. Moreover, it indicates the potential for extending this approach to other object types, such as pedestrians and cyclists, by appropriately fitting the model to relevant data.

**Figure 12:**
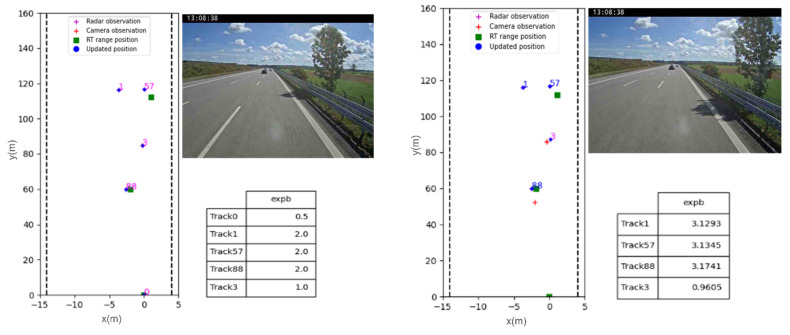

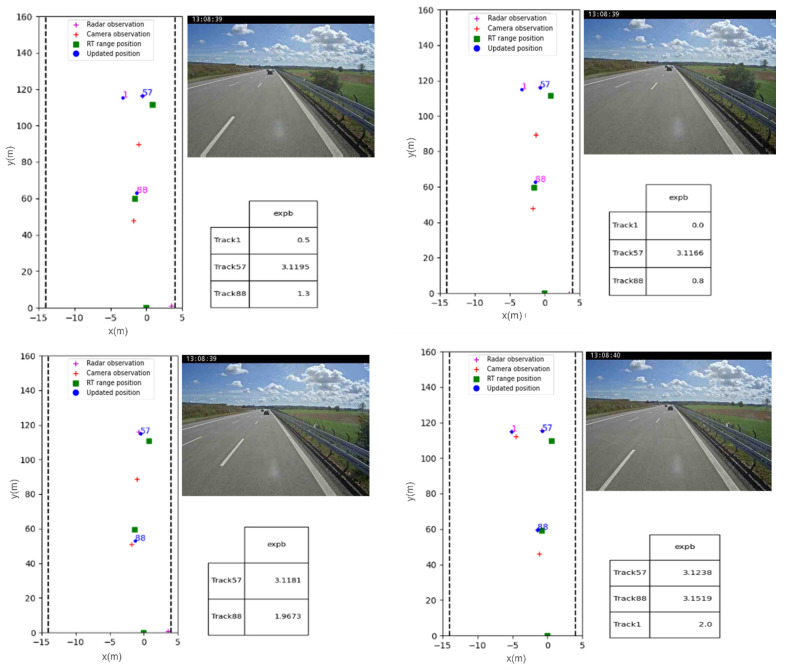
A highway tracking scenario with tracking results and table of existence scores. First, all the tracks are in magenta with ‘hidden’ status, then sensor-only tracks (Track (3) and Track (88) later on) and occluded track (Track (1) where both sensors observations were lost) have their status shifted to ‘hidden’.

**Figure 13:**
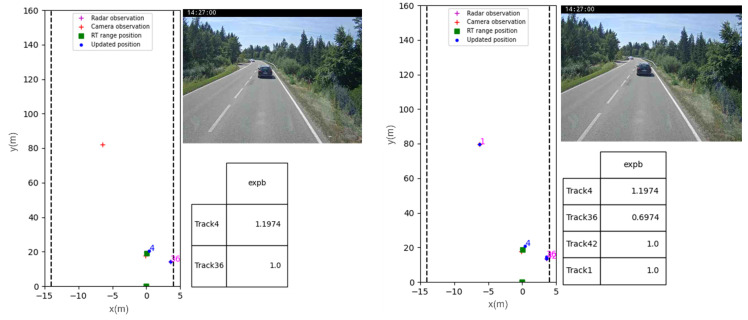

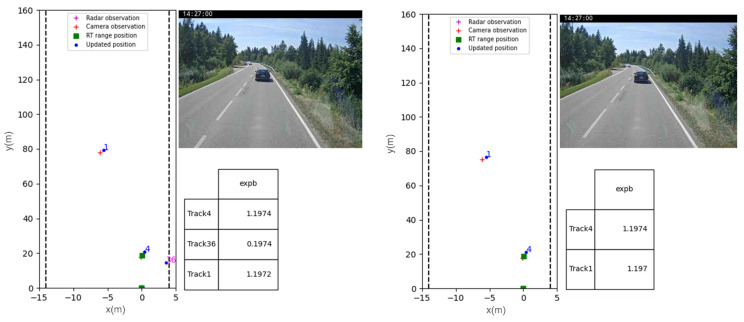
Frames extracted from peri-urban tracking scenario of two cars. Only the car in ego lane is equipped with an RT range ground truth system. Track (36) being a radar-only false positive, is being correctly deleted from the tracking scenario because of a decreasing existence score.

In Figure 12 and Figure 13, we present two tracking scenarios in highway and sub-urban environments. The figures use color annotations to indicate the visibility status of each object. When an object is ‘hidden’, its ID is written in magenta, and when it is ‘visible’, the ID is written in blue. In the first frame, the object ID annotations are in magenta for all three tracks because their existence scores do not satisfy the validity threshold, which we set empirically to 1.5. The existence score of an object increases if it is detected and tracked over time until it becomes higher than the validity threshold, at which point the ID color switches to blue.

However, in Figure 12, Track (3) remains ‘hidden’ because it is a camera-only track and its existence score remains lower than the validity threshold. Meanwhile, Track (1) color turns magenta when it becomes unobservable by both the camera and the radar. This occurs due to occlusion by Track (88). As a result, its existence score continues to decrease until it reaches zero, leading to its deletion from the tracking scenario. A comparable situation is observed with Track (88). When not detected by the radar but only by the camera, its existence score continues to decrease. However, once the radar starts detecting Track (88) again, its status shifts to ‘visible’, as shown in Figure 12.

In Figure 13, two observed cars initialized with a ‘hidden’ state. In the subsequent frame, these objects are validated, and the tracking algorithm begins to track Tracks (1) and (4). It is important to note that only the heading vehicle is equipped with an RT-range system, while the second oncoming car is observed by the MFC (Multi-Function Camera). Additionally, in this figure, an example of how false positive detections are handled is presented. An initial existence *ES = 1.0* score is assigned to Track (36) originating from a radar false positive detection. Then, the existence score gradually decreases because of unstable radar-only observations, ultimately leading to the deletion of Track (36).

### 3.2. Discussion

Compared to a classical Kalman filter, the LSTM correction model shows much better results in highway and peri-urban environments. The Kalman filter’s limitation in modeling observation errors that increase with distance becomes apparent in challenging scenarios. Conversely, our model adeptly adapts to this behavior, making it particularly valuable for our Multi-Function Camera (MFC) when estimating the distances of detected objects, where errors grow with distance.

Additionally, radar detections are vulnerable to clutter from multipaths and reflections, leading to a higher rate of false positives. Our association management utilizes an intelligent existence score to address these sensor-related challenges, which accounts for association scores of a track with both the sensor’s observations and for the number of skipped frames. This track management effectively reduces the influence of individual sensor observations, thus bolstering the overall tracking performance.

By breaking down the tracking process into manageable components and training individual models for each step, we were able to focus on specific aspects of the tracking problem. This approach allowed us to fine-tune the models for their respective tasks, optimizing their performance for specific challenges. Consequently, we achieved better results in scenarios characterized by complex dynamics, occlusions, and sensor-specific limitations.

Furthermore, the training of individual models demanded a smaller training dataset compared to a single monolithic model. As a result, we mitigated the burden of gathering and annotating a large amount of training data, which can be particularly challenging and expensive in the context of multi-object tracking. Instead, our approach allowed us to make efficient use of available data and yielded promising results even with a more limited dataset.

However, the selection and distribution of learning data play pivotal roles in DL-based approaches. It is useful that these datasets are thoughtfully curated to encompass a comprehensive spectrum of scenarios and dynamic sequences. Furthermore, in the context of correction models, it is important to acknowledge that these models are designed to predict errors specific to individual sensors. Consequently, a distinct model must be trained for each sensor to ensure accuracy and effectiveness. Therefore, a challenge emerges when the inherent characteristics of a sensor undergo modifications, as this necessitates an adjustment in the training approach.

## 4. Conclusions

Despite the significant progress in recent studies, multi-object tracking remains a challenging subject due to complexities introduced by object motion, interactions, and occlusions. Imperfect sensor detections, including false alarms, missed detections, and noisy readings, add further complications.

To address these challenges, we proposed an LSTM-based multi-object tracking solution, utilizing three trained models for data association, track update, and predictions. Our approach simplifies the training process, and the LSTM models with simple architectures were trained on a database collected from both highway and peri-urban environments.

Our results demonstrate that the LSTM models learned association metrics that outperformed the classically used Euclidean or Mahalanobis distances, providing more representative track-observation relationships.

For predicting sensor errors, the LSTM-based models showed promising results compared to the Kalman-updated estimations, accurately learning the sensors’ observation models. However, we acknowledge that further improvements can be achieved in the prediction of the state vector and in predicting the trajectory of a track by using a larger training database and deeper models to learn object dynamics better. Furthermore, we proposed a track management process using an existence score that integrates the association scores. The existence score is variable and is computed at each iteration for each track in order to efficiently handle the death and birth of the tracks but also to reduce false positives and missed detections.

In future work, we aim to address object interactions, which are crucial in predicting future state vectors, especially in crowded scenes for autonomous vehicle applications. Considering interactions will enable better predictions of object movements based on their neighborhood and environment. One potential approach is to input multiple close tracks and their trajectories into the prediction model to make simultaneous predictions for all neighboring objects.

Overall, our LSTM-based approach shows promising results in multi-object tracking and lays the foundation for addressing more complex scenarios in the future.

## Figures and Tables

**Table 1:** Comparison of our LSTM-based method with Kalman-based tracking.

	Kalman	Our Proposed Algorithm
Sensor model	Yes	No
Dynamic model	Yes	No
Needs learning	No	Yes
Estimation update accuracy	0.7 m	0.09
Computational complexity	Low	Moderate
Versatility	Restricted	Wide range of scenarios

**Table 2:** Association error and MOTA error.

Error Type	% Association Model	% Euclidean Distance
False Positives	2.12%	3.45%
Misses	0.17%	0.15%
Mismatches	2.60%	4.11%
MOTA	95.10%	92.30%

## Data Availability

Data are contained within the article.
